# Phytogenic Ingredients from Hops and Organic Acids Improve Selected Indices of Welfare, Health Status Markers, and Bacteria Composition in the Caeca of Broiler Chickens

**DOI:** 10.3390/ani11113249

**Published:** 2021-11-13

**Authors:** Monika Michalczuk, Elisabeth Holl, Anne Möddel, Artur Jóźwik, Jan Slósarz, Damian Bień, Katarzyna Ząbek, Paweł Konieczka

**Affiliations:** 1 Department of Animal Breeding, Institute of Animal Sciences, Warsaw University of Life Sciences, Ciszewskiego 8, 02-786 Warsaw, Poland; monika_michalczuk@sggw.edu.pl (M.M.); jan_slosarz@sggw.pl (J.S.); damian_bien@sggw.edu.pl (D.B.); 2 Dr. Eckel Animal Nutrition GmbH & Co. KG, Im Stiefelfeld 10, D-56651 Niederzissen, Germany; e.holl@dr-eckel.de (E.H.); a.Moeddel@dr-eckel.de (A.M.); 3 Institute of Genetics and Animal Biotechnology, Polish Academy of Science, Jastrzębiec, Postępu 36A, 05-552 Magdalenka, Poland; aa.jozwik@igbzpan.pl; 4 Department of Animal Nutrition, The Kielanowski Institute of Animal Physiology and Nutrition, Polish Academy of Sciences, Instytucka 3, 05-110 Jabłonna, Poland; k.zabek@ifzz.pl; 5 Department of Poultry Science, University of Warmia and Mazury in Olsztyn, 10-719 Olsztyn, Poland

**Keywords:** broiler chicken, phytogenics, organic acids, production results, gut health, welfare indices

## Abstract

**Simple Summary:**

The selection for the rapid growth rate in broiler chickens that has been carried out over the years has negatively influenced their health and welfare status. In recent years, a number of reports have been delivered on the use of additives that improve broilers’ intestinal peristalsis and production results. The authors of this paper have proved that applying a mixture with 50% hops (manifesting strong antioxidant, antibacterial, and antifungal properties) may bring benefits to the quantity and quality of the final product. This may refer to the production performance, flock health status, and welfare of birds. The thematic scope of this research is currently of significant importance, as veterinary inspections pay particular attention to the quality of litter and the welfare of birds, and this motivates producers to improve breeding conditions, which will contribute to better production systems.

**Abstract:**

The objective of this study was to determine the influence of phytogenic product-supplemented, organic acid-supplemented, and prebiotic-supplemented diets on the production results, antioxidative status, and selected welfare indices in broiler chickens. A total of 1155 one-day old male Ross 308 broilers were randomly assigned to one of three treatment groups: Group C, no additives; Group A, supplemented with phytogenic supplement (50% hop); and Group P, supplemented with 65% organic acids and their salts, and 30% prebiotic complex. Health condition and production results were monitored during the entire experiment. After 42 days, 10 birds from each dietary treatment group were selected for blood sampling and slaughter analysis. The results obtained revealed that over the whole feeding period, none of the investigated additives significantly affected broiler performance indices. However, feeding the birds treatment-A increased the relative abundance of *Bifidobacterium* in caecal digesta compared to the other treatments, whereas feeding treatment-P increased the relative abundance of *Lactobacillus* compared to the control treatment. Overall, treatment-A was more effective at increasing relative abundance of *Clostridia* in birds at 42 days of age than treatment-P. Finally, there were no changes in blood levels of antioxidant indices or liver function indicators.

## 1. Introduction

Legal regulations state that since 2006, the preventive use of antibiotics and antibiotic growth promoters (AGP) in farm animals is banned in the European Union; therefore, studies on the use of plant preparations as drug alternatives in the diets of chickens have been carried out [1,2,3]. This is because, in the intensive broiler industry, the prevalence of compromised bird health, particularly that associated with the gut’s functional status, has increased due to the withdrawal of AGP. The functional status of the gut, which determines to a great extent not only the birds’ health but also the conditions of the chicken coop, depends on several factors, including the integrity of gut epithelium, dietary composition, and the commensal microbiota [4]. Overall, the barrier integrity in birds can be compromised by either a non-infectious method, such as feeding programs, dietary imbalances, dietary non-starch polysaccharides and protein levels, or feed contamination (e.g., mycotoxins), or by infectious factors including pathogen challenge [5,6,7]. These complex interactions between the host and the factors that affect birds’ health have created a great challenge for the poultry sector in developing reliable, cost-effective alternatives that are fully integrated with the production system in order to improve the birds’ health while maintaining good production results [8]. Moreover, consumer concern about the ways food animals are raised has created a move by producers and the industry towards transparency and has also led to demands for the use of antibiotic and AGP alternatives in the production setting [6,7,9].

The market for feed additives has developed greatly in recent years and has adapted its offer to the needs of manufacturers. Feed additives, which are used in poultry diets, consist of probiotics, prebiotics, acidifiers, medium-chain triglycerides, and herbal extracts. Preparations based on herbs are increasingly popular due to consumer preferences and the search for natural products. In recent years, the term “phytogenics” has appeared in the market—meaning, preparations of plant origin obtained from herbs containing bioactive substances of secondary metabolism [10,11]. Secondary metabolites are the products of specialised metabolisms, which are indispensable for the survival of plants, and include the following compounds: glycosides, alkaloids, flavonoids, terpenes, essential oils, pectin, and plant mucoid fluids as well as organic acids, vitamins, and mineral salts. Phytogenics, due to their high content of biologically active components, appear to be very promising in terms of a prophylaxis for poultry. However, one of the factors that is of key importance is production profitability, which warrants that the application of the treatment must fulfil economic criterion to be successful, particularly in commercial conditions. In this regard, the recommendation is that phytogenics must be administered throughout the whole production period to be efficient for prophylaxis. According to calculations, the cost of the phytogenic prophylaxis is typically equivalent to the administration of antibiotics to a flock for a period of five days [12].

The welfare indices, in particular the leg condition and walking ability of broilers reared in intensive conditions, is strongly influenced by the coops’ condition [13]. This association can be explained due to the direct influence of feed on the gut’s microbial composition and activity, and the utilisation of the feed’s ingredients by the birds, which determines, in consequence, the conditions of the chicken coop (by the quality of the litter) [14]. Birds reared under unfavourable conditions are exposed to an increased prevalence of walking difficulties, lesions, and/or depressed growth [15]. Currently, the economic loses in broiler production caused by leg weakness and footpad dermatitis (FPD) have been identified as one of the major challenges in the poultry production sector [16,17]. FPD has been defined as an important problem in the modern poultry industry and negatively affects the birds’ welfare and health status through compromised walking and feeding activity as well as compromised growth performance. For example, in the Netherlands, almost 38% of broilers have been reported as having severe footpad lesions, while 26% had mild footpad lesions, indicating, among other things, a problem of magnitude [18].

The present study aimed to investigate the potential of using phytogenic ingredients from hops, liquorice, and gum arabic, or a mixture of a free butyrate acidifier and gluconic acid and a prebiotic complex (the prebiotic component in PreAcid is gluconic acid, 20% in the form of calcium gluconate) to improve broilers’ welfare. The assessment was based on the broilers’ walking ability, the skin condition of foot pads, and selected indices of antioxidant status and performance results in semi-commercial conditions.

## 2. Materials and Methods

### 2.1. Birds, Diets, and Experimental Design

The trial was conducted on a total of 1155 one-day-old male Ross 308 broiler chickens. The birds were randomly assigned to one of three treatment groups (five repeated pens for each group, with 77 chicks per pen; *n* = 5): Group C, without any supplements; Group A, supplemented with a phytogenic product based on more than 50% hop ingredients in combination with a premix of liquorice, gum arabic, and other plant ingredients (13%); and Group P, supplemented with 65% organic acids and their salts (formic and lactic) and 30% prebiotic complex (the prebiotic component was gluconic acid, 20% in the form of calcium gluconate) [19].

The diets were commercial, and each feeding was formulated to meet or exceed the requirements for Ross 308 broiler chickens. The amounts of supplement included in Group A were as follow: 400 mg/kg in the starter, 300 mg/kg in grower I, and 200 mg/kg in grower II and the finisher. The supplement amounts included in group P were: 3 g/kg in the starter, 2 g/kg in grower I, and 1 g/kg in grower II and the finisher. Overall, each treatment was repeated for five pens of 77 chickens each. Lighting and temperature in the building were maintained according to the Ross Management Guide [20]. The one-day-old chickens were vaccinated at the hatchery against infectious bronchitis, Gumboro disease, Marek’s disease, and Newcastle disease. All broilers were individually weighed (BW) throughout the experiment period on days 1, 10, 24, 35, and 42. Likewise, when diet changes took place, the total feed consumption for the respective feeding phases for the periods 1–10, 11–24, 25–35, and 36–42 days was determined for each group on a per pen basis. Based on these data, the feed conversion ratio (FCR) was calculated for the respective experimental periods (total weight of feed consumed/obtained gain). Mortality was recorded during the whole trial period. The European Yield Coefficient (EYC) was calculated for the whole period of rearing (42 days). EYC was calculated based on the following formula:

$$EYC=\frac{BW\times livability\#\;\left(\%\right)}{day\;of\;production\times FCR\;\left(\frac{kg}{kg}\right)}\;\#livability\;is\;characterized\;by\;the\;ratio\;of\;the\;final\;number\;of\;birds\;to\;the\;initial\;number.\;$$

During the experimental period, the broilers were fed according to the following feeding program: starter, days 1–10; grower I, days 11–24; grower II, days 25–35; and finisher, days 36–42. The birds had ad libitum access to feed and water consumption. The composition of the diets was determined using the standard AOAC method [21] (Table 1).

### 2.2. Litter Analysis

Litter samples were collected once every week from five different sites (two samples of the horns and around water lines, around feeder lines, and around the free area) within each coop and pooled and mixed before being measured (50 g). The litter moisture content was determined by the weight loss on drying. In brief, the pooled litter was weighed, then dried for 12 h at 105 °C in an electric drying oven (Model: WAMED, SUP–100 W5, Warsaw, Poland), and then weighed again. The decreased weight was recorded as dry matter (%).

### 2.3. Gait-Scoring Observations

Assessment of walking ability and foot pad condition. The birds’ ability to walk was scored on a six-point scale at the thirty-ninth day of life and was performed by qualified veterinarian staff. The following detailed descriptions of each gait score were applied, but not all the attributes of a score were necessarily identified in each bird [22]. One hundred and fifty birds were examined from each group, selecting randomly 30 from each pen, according to following scale.

Gait score 0. The bird walked normally with no detectable abnormality; it was dexterous and agile. Typically, the foot was picked up and put down smoothly and each foot was brought under the bird’s centre of gravity as it walked (rather than the bird swaying). Often, the toes were partially furled while the foot was in the air. The bird should have been capable of balancing on one leg and walking backwards easily if necessary. It should also have been in full command of where it was going and been able to easily deviate from its course to avoid other birds.

Gait score 1. The bird had a slight defect that was difficult to define precisely but would have precluded its use for breeding if gait had been the sole selection criterion at the standard of a pedigree breeder. For example, the bird may have taken unduly large strides which, although the observer may not have recognised the exact cause, produced an uneven gait.

Gait score 2. The bird had a definite and identifiable defect in its gait, but the lesion did not hinder it from moving or competing for resources. For example, it may have been sufficiently lame in one leg to produce a rolling gait that did not seriously compromise its manoeuvrability, acceleration, or speed.

Gait score 3. The bird had an obvious gait defect that affected its ability to move about. For example, the defect could take the form of a limp, a jerky or unsteady strut, or a severe splaying of one leg as it moved. The bird often preferred to squat when not coerced to move, and its manoeuvrability, acceleration, and speed were affected.

Gait score 4. The bird had a severe gait defect. It was still capable of walking, but only with difficulty and when driven or strongly motivated. Otherwise, it squatted down at the first available opportunity. Its acceleration, manoeuvrability, and speed were all severely affected.

Gait score 5. The bird was incapable of sustained walking on its feet. Although it may have been able to stand, locomotion could only be achieved with the assistance of the wings or by crawling on its shanks.

The gait scoring was performed by two people, one gently herding and driving each bird with a light cane, and the other observing from a crouched position. The two assessors had different views of the bird, and agreement between them for each bird was required before the score was recorded. In some cases, it was necessary to assess a bird over several passes, each typically of about 2 m.

### 2.4. Foot Pad Dermatitis Observation

Foot pad condition was visually defined five days before slaughter according to a five-step scale [23]. The veterinarians’ evaluation consisted of determining the degree of the lesions’ advancement on a scale of 0, 1, or 2 (Table 2). All birds involved in the experiment were subject to evaluation.

### 2.5. Sampling Procedures

Thirty chickens were chosen (10 birds from each treatment) for slaughter at the age of 42 days of life that had a body weight similar to the group mean. Blood samples were collected from the jugular vein for biochemical and antioxidant analysis.

We aimed to evaluate the relative abundance of selected bacterial groups in caeca. On day 42 of age, a total of 10 chickens from each dietary treatment group were selected, representing the mean group by BW, and the luminal contents of both caeca from each bird were collected in sterile tubes and immediately frozen at −32 °C for further analysis. To determine the relative abundances of bacteria in the caecal samples, the following previously described protocol was applied [24]: briefly, the caecal digesta was thawed at 4 °C for four hours, and then, bacterial genomic DNA was extracted from digesta (approximately 200 mg) using the QIAamp Fast DNA Stool Mini Kit (Qiagen, Hilden, Germany) according to the manufacturer’s protocol. The yield and purity of the isolated DNA were then estimated spectrophotometrically (Nanodrop, NanoDrop Technologies, Wilmington, DE, USA). The primer sets that were used for determining the respective bacterial populations are presented in the Table 3. The PCR conditions were applied as reported above for each respective bacteria group. The obtained PCR-products were separated by electrophoresis on a 2% agarose gel. PCR products were quantified using ImageJ 1.47v software for densitometry measurements, National Institute of Mental Health, Bethesda, MD, USA (NIMH), with the density of the bands for each of bacteria group expressed in relation to the density of the total bacteria primer product. Each sample was analysed in duplicate.

Chemical analysis. Blood biochemical parameters such as enzymes (aspartate transaminase—AST, EC 2.6.1.1; alanine transaminase—ALT, EC 2.6.1.2; gamma-glutamyltransferase—GGT, EC 2.3.2.2) and electrolytes (sodium—Na, potassium—K, chloride—Cl, and calcium—Ca), glucose—GLU, magnesium—Mg, phosphorus—P, lactate (LAC), and total protein (TP) were determined using a Cobas INTEGRA 400 Plus biochemical analyser (Roche Diagnostics, Basel, Switzerland) based on spectrophotometry, turbidimetry, fluorescence polarisation, and ion-selective potentiometry methods. Samples of blood were collected in sterile tubes without an anticoagulant. To obtain serum, whole blood was centrifuged at 2000× *g* for 10 min at 4 °C and then placed in analyser racks.

The level of vitamin C in the serum was determined using a LambdaBio-20 spectrophotometer (Perkin Elmer, Waltham, MA, USA). The following components were mixed thoroughly and centrifuged for 20 min: 0.5 mL of tissue homogenate, 0.5 mL of distilled water, and 1.0 mL of 10% trichloric acid. The obtained supernatant at 1.0 mL was combined with 0.2 mL of 2,4-dinitrophenylhydrazine-thiourea-copper sulphate reagent and incubated at 37 °C for 2 h. Subsequently, 1.5 mL of 65% sulfuric acid was added, and the mixed sample remained at room temperature for another 30 min. The change in colour of the sample was measured at 520 nm. Solutions of vitamin C standards (0.5–5 mg of vitamin C A92902 Sigma-Aldrich L-Ascorbic acid 99%) were treated similarly.

Measurements for radical scavenging activity were performed by routine assay procedure [29] using a synthetic DPPH radical (1,1-diphenyl-2-picrylhydrazyl). Folin–Ciocâlteu reagent was used as an oxidizing reagent, and all the chemicals were purchased from Sigma-Aldrich Chemie GmbH (Munich, Germany) in the highest available purity.

Glutathione (GSH) concentration was determined in the whole blood by means of the OxisResearch™ Bioxytech^®^ GSH/GSSG—412™ test (Foster City, CA, USA). Before the analysis, the samples were frozen with the addition of M2VP (1-methyl-2-vinyl-pyridium trifluoromethanesulfonate) at a temperature of −80 °C. The released, reduced GSH was determined in accordance with the detailed instruction provided by the kit’s producer. The absorbance reading (λ412) and the measurement of reaction kinetics were performed using the microplate reader Synergy 4 (BioTek; Winooski, VT, USA). The results were calculated using Gen5 software (BioTek). GSH concentration was expressed in thiol groups (mmol-SH groups).

The determination of the content of total phenols was performed as previously described [30].

The samples were thoroughly mixed, and after 8 min, 2 mL of saturated sodium carbonate solution was added. The next stage of the analysis involved an incubation test at 40 °C for 30 min (until a stable characteristic blue colour had developed). The absorbance was measured at 765 and 735 nm against a blank sample (experimental material replaced with 0.5 mL ddH_2_O). Results were read using a calibration curve plot based on the absorbance of a gallic acid standard in the range of 0–0.5 mg/mL and expressed in mg gallic acid equivalent (GAE/mL) serum.

### 2.6. Statistical Analysis

The statistical analysis was performed using SPSS 23.0 for Windows. Analysis of variance was used to determine the influence of experimental factors on production results, antioxidative status, and the welfare of the broiler chickens. The normality of the variables’ distribution was checked using the Kolmogorov–Smirnov test. In turn, the Kruskal–Wallis test was used to determine the effect of treatment on body weight at each term of analysis, while the Mann–Whitney test was used to determine the differences between groups. Finally, the Chi-square test was employed to estimate the frequency of gait scoring and FPD in particular groups.

### 2.7. Ethical Statement

All procedures in the present study were performed in accordance with the principles of the European Union and Polish Law on Animal Protection. This study was conducted by qualified veterinarians who performed all procedures that involved the handling of the birds. No action involving pain or suffering was practiced. This study was run in accordance with Directive no. 2010/63/EU and did not require the approval of the Local Ethics Committee based on the regulation of the Ethic Committee of November 2019 (resolution no. 174/2019).

## 3. Results

### 3.1. Performance Response

Overall, there were no significant differences in the production results for the chickens that were given the treatments over the entire 42-day feeding period. Numerically, the best final body weight was achieved by chickens from group A (3.46 kg) and the lowest from group P (3.43 kg). Numerically, the best FCR result was also seen in group P, 1.49 kg/kg. The European Yield Coefficient (EYC)—which considers growth performance expressed by BW, feed efficiency expressed as the feed conversion ratio (FCR), the production duration over the 42 days, and the mortality rate during this period—was used to assess the economic efficiency of the chickens. The number of points that produced an effect in the treatment-A group was 532 points, group P—522 and group C—518.

### 3.2. Walking Ability and Foot Pad Condition

**Figure 1:**
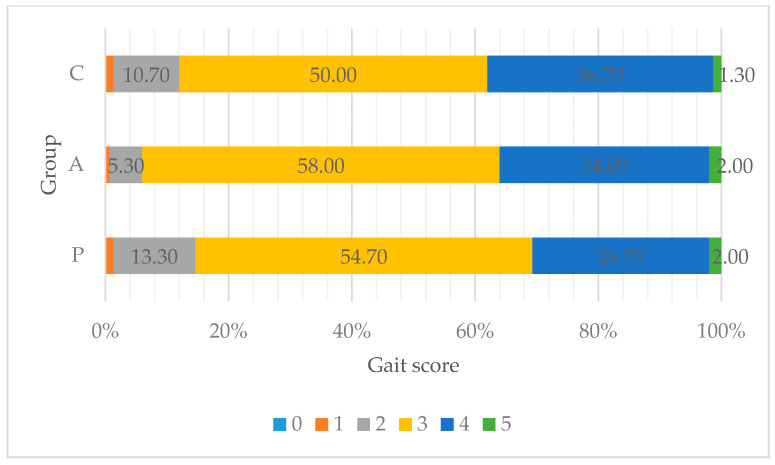
Assessment of walking ability in broiler chickens (%). The birds were fed a non-supplemented control diet (C); the control diet supplemented (in addition) with a phytogenic supplement containing hop (A); or the control diet supplemented with organic acids and their salts and prebiotic complex mixed in the proportion of 65/30 *v/v*, respectively (P). A gait score of 0 indicated the best walking ability, whereas 5 indicated the worst walking ability (a detailed description of respective gait scores can be found in the Materials and Methods section). The variation between groups did not differ significantly (*p* = 0.346). For the studied groups, the median was 3, which means that over half of the birds were evaluated as 3. Mean + SD: C = 3.26 ± 0.718; A = 3.31 ± 0.636; P = 3.17 ± 0.730. A total of 30 birds from each replicate (*n* = 5) was evaluated.

**Figure 2:**
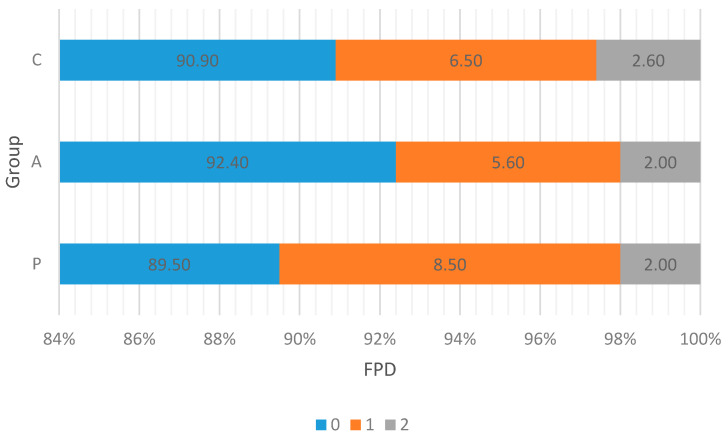
The occurrence of foot pad dermatitis in broiler chickens (%). Birds were fed a non-supplemented control diet (C); the control diet supplemented (in addition) with a phytogenic supplement containing hop (A); or the control diet supplemented with organic acids and their salts and prebiotic complex mixed in the proportion of 65/30 *v/v,* respectively (**P**). A gait score of 0 represented a lower frequency of foot pad dermatitis occurrence, whereas 2 represented a higher frequency of footpad dermatitis occurrence. The variance between groups did not differ significantly, *p* = 0.275. For the studied groups, the median was 0, which means that over half of the birds were evaluated as 0. Mean ± SD; C = 0.12 ± 0.393; A = 0.1 ± 0.393; P = 0.13 ± 0.387. All birds were evaluated.

The data obtained regarding walking ability and leg conditions revealed that numerically, the best gait indicating walking ability was observed in the group of birds administered with treatment-P (the best walking ability was 13.3% vs. 10.7% and 5.3% for C and A treatments, respectively) (Figure 1). Where FPD occurred, the data indicated that there was no difference between either experimental treatment regarding a higher frequency of lesion occurrence (2%), but in the control group, it was numerically higher by 0.6 percentage points, yet the result has not been statistically proven (*p* = 0.275) (Figure 2).

### 3.3. Determination of Litter Dry Matter

**Figure 3:**
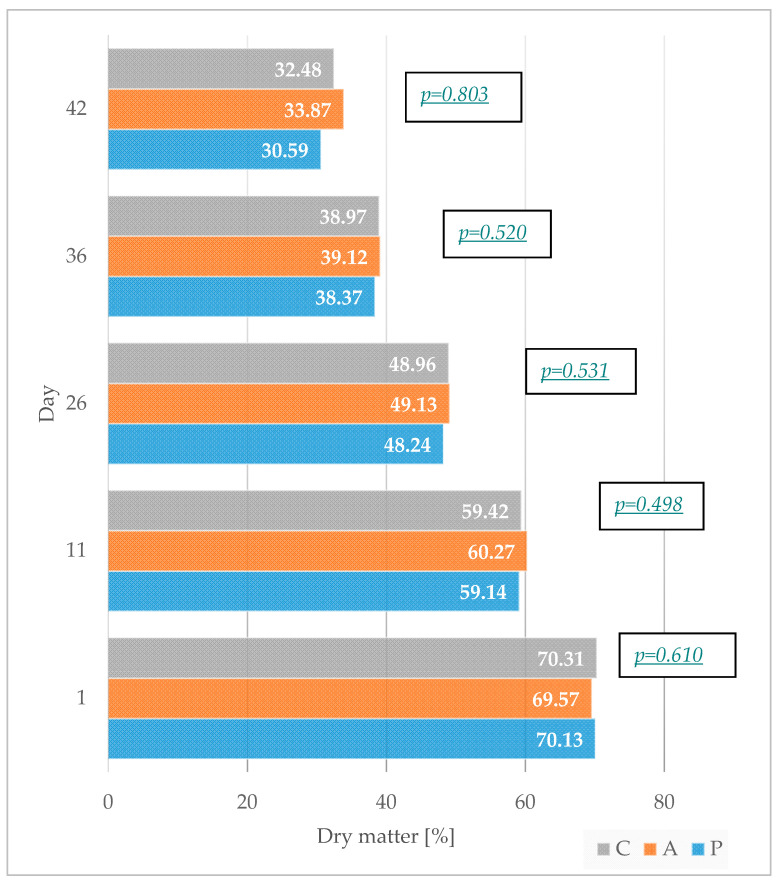
Dry matter content in the litter at day 1, 11, 26, 36, and 42 of the rearing period. Birds were fed a non-supplemented control diet (C); the control diet supplemented (in addition) with the phytogenic supplement containing hop (A); or the control diet supplemented with organic acids and their salts and prebiotic complex mixed in the proportion of 65/30 *v/v,* respectively (P).

The assessment of dry matter content in the litter is presented in Figure 3. Overall, the dry matter content for each group decreased consistently over the 42-day period, with averages of 70% on day 1 and 32.3% on day 42.

### 3.4. Bacterial Composition in the Caecal Digesta

**Figure 4:**
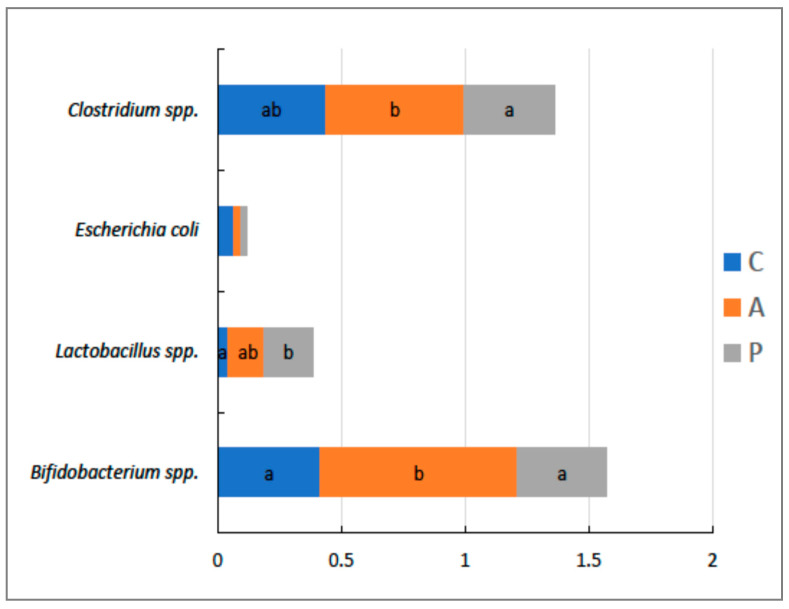
The effects of dietary treatments on the relative abundance (in relation to the density of total bacterial primer product) of selected bacteria in the caecal digesta of chickens at 42 days of age. Birds were fed a non-supplemented control diet (C); the control diet supplemented (in addition) with the phytogenic supplement containing hop (A); or the control diet supplemented with organic acids and their salts and prebiotic complex mixed in the proportion of 65/30 *v/v,* respectively (P). Data are the means of 10 replicates per treatment. The ab means within columns without a common superscript differ significantly (*p* < 0.05).

The dietary treatments significantly affected the relative abundance of selected bacteria in the caecal digesta of the birds at 42 days of age (Figure 4). Feeding the birds treatment-A resulted in a significantly higher relative abundance of Bifidobacterium than in the other treatments (*p* = 0.023), whereas feeding the birds treatment-P increased the relative abundance of Lactobacillus compared to the control treatment (*p* = 0.045). Overall, it was evidenced that treatment-A was more effective at increasing Clostridia relative abundance in birds at 42 days of age than treatment-P (*p* = 0.028).

### 3.5. Blood Indices of Metabolism

The metabolic profile parameters were determined for the blood of the birds at 42 days of life. It was shown that the activity of the determined liver enzymes, for example, AST and ALT, were not significantly different for the two treatments. The GGT activity was significantly (*p* < 0.05) higher in the P and A groups than in the control. We also evidenced significant changes in LAC activity as well as K and P concentrations in the blood. In the control group, all these parameters were significantly higher (*p* < 0.05) than in the remaining groups. Other biochemical parameters analysed, such as glucose, total protein, and Ca, Mg, Na, and Cl, did not differ between the treatments (Table 4).

The analysed antioxidant indices in the blood showed that there were no differences in indices such as GSH (1.073, 1.175, and 1.14 mmol–SH; C, A, and P groups, respectively), percentage of DPPH (82.57%, 79.69%, and 83.34%), total polyphenols (2.30, 2.29, and 2.27 mg GAE/mL), and vitamin C (9.01, 8.75, and 8.43 mg/100 mL) in the birds as a result of the dietary treatments. Altogether, the blood-indices’ response showed that the use of the investigated feed additives did not disturb the oxidative status of the birds as assessed using blood markers.

## 4. Discussion

The incidence of leg problems such as FPD are currently considered leading welfare issues in poultry farming. FPD is characterised by inflammation and necrotic lesions (which can be superficial through to deep) on the plantar surface of footpads and toes [31]. The origin of FPD is quite complex as it is believed to result from several factors, but is particularly related to litter moisture, an imbalance in nutrition, and genetic susceptibility [16]. However, there is speculation that the primary contributor to a susceptibility to FPD is related to the quality of bedding material, which is closely related to its moisture content and microbial characteristics, particularly in the last period of birds’ growth [32]. Therefore, in the current study, both walking ability and FPD assessments were done in the last stage of the rearing period, when the birds are the most susceptible to locomotive disorders due to their high BW and poor litter conditions [17]. In this regard, there were no statistically significant differences in the walking ability and FPD parameters between the control and treatment groups. Despite this, however, a possible cross-link between the dietary treatments and litter quality could be implied due to the changes observed in bacterial composition of caecal digesta, which to a great extent contributed to the overall litter conditions. In line with this, the most effective treatment in the present study that caused a shift in the amounts of caecal bacteria was the treatment supplemented with phytogenics because in the birds fed with this treatment, the relative abundance of Clostridia was significantly higher than in the group fed the organic acid supplemented diet; the same was true regarding the relative abundance of Bifidobacterium. On the other hand, feeding the birds a diet supplemented with organic acids resulted in a significant shift in the relative abundance of Lactobacillus in the caecal digesta compared to control. The Lactobacillus species reside in the caeca and are present in great abundance, as well as playing a key role in the process of fermentation of the substrates that reach this environment. These bacteria are also responsible for the production of lactic acid, which manifests regulatory properties on gut function [33]. According to another report [34,35], some pathogens, including Clostridium perfringens, belonging to the group Clostridia, are known to be responsible for the skin inflammation that causes necrotic lesions in birds; however, due to the fact that in the present study, the investigated supplements did not compromise foot pad condition, walking ability, or the performance response of the chickens, we excluded the probability that the proportion of these pathogens increased along with Clostridia shift as a result of the applied treatments. Because the Clostridia group are involved in the metabolism of short-chain fatty acids, which are a major end-product of bacterial fermentation, they therefore determine the properties of the digesta and excreta [36]. This was also confirmed in our preliminary study [19], in which higher butyric acid concentration in the caecal digesta was evidenced as a result of organic acid supplementation. Given this, we may speculate that the changes in the abundance of key groups of bacteria in the caecal digesta, which are beneficial to the host, were responsible for the improved litter quality and skin condition of the birds’ legs. This is in line with our preliminary study, in which evidence was found that the supplementation of broiler diets with either phytogenic compounds or organic acids significantly affected caecal bacterial activity (assessment based on short-chain fatty acid production), indicating a possible association [19]. The beneficial role of the Bifidobacterium group in modulating digesta properties in farm animals has also been well reported [37]. The investigated additives’ possible mode of action in the present study could have been manifested through indirect action via short-chain fatty acids and lactic acid, which can penetrate the microbial membrane and dissociate into protons and anions, thus acidifying cytoplasm and making sensitive bacteria dysfunctional and unable to maintain optimal pH, which, in turn, results in changes in their abundance in the caecal environment [38]. In this study, we investigated the effect of phytogenic additives and organic acids on the microbiota composition within the caecal digesta instead of litter/faeces, as the indices for litter/faeces varies greatly depending on the coops’ conditions. According to Williams and Athrey [39], even cloacal swabs have been shown to be an unreliable source for reflecting the gut microbiota community and structure in birds due to inter-individual variation and high degrees of randomness. We link the possible action of both the investigated additives with their likely influence on microbiota composition in the caeca as was reported in our preliminary study regarding microbiota activity [19]. However, more insights in this regard are needed.

The determination of the blood indicators of metabolism by laboratory analysis is an important procedure that assists in the diagnosis of various avian diseases and disorders. The response of the animal’s body is investigated on the basis of the enzymatic activity responsible for the hepatic profile of gamma-glutamyl transferase (GGT), aspartate aminotransferase (AST), and alanine aminotransferase (ALT) as well as the electrolytes C, K+, Na+, and phosphor [38]. For the sake of animal health and welfare, increasing attention has now been given to oxidative stress parameters [13,40]. It is already known that the occurrence of dietary-induced oxidative stress is correlated with the induction of inflammation markers. This may indicate impaired immunity, which may affect skin quality, increase FPD risk, and consequently lead to a deterioration in the welfare and health status of the birds. Because blood indices reflect the physiological status of the birds, they can be used as indicators of the birds’ health, nutrient metabolism, and physiological status. Chickens that suffer from metabolic disorders can be diagnosed based on liver function blood markers, including ALT, AST, and GGT [31]. The lack of changes in AST and ALT levels for the treatments reported in this study may suggest that the supplements administered in the study (either P or A) did not cause metabolic changes that affected liver function when compared to the control group. In contrast, the observed changes in GGT activity were rather marginal for the birds’ physiological status since no significant differences in growth rate was observed. The increased GGT levels for the P and A treatments may also suggest intensified liver workload; however, no deterioration in the health status of the birds was observed, and the mortality levels remained within acceptable norms [41]. Liver load was not significant for the birds in the study groups because we observed a lower concentration of lactate in their blood. Blood lactate increases as a result of physical stress and severe tissue hypoxia, or respiratory failure. Rising levels can lead to metabolic acidosis. The lower value of lactate in the blood of these birds may also indicate a reduction in energy requirements related to physical activity. In the present study, lower lactate activity was confirmed in both experimental treatments and indicates that neither additive disturbed the oxygen supply. Oxygen delivery may have indicated reduced gait movement resulting from high body weight at 42 days of age in groups P and A. Broilers in commercial settings are exposed to a range of stressors. A growing body of information clearly indicates that excess, reactive oxygen and nitrogen species (ROS/RNS) production, and oxidative stress are major detrimental consequences of the most common commercial stressors in poultry production [41,42]. Our data indicate that the differences reported for the selected blood indices in the birds were likely not associated with any physiological disorders. This is also supported by the performance results reported in the present study, which showed no significant differences due to dietary treatments, indicating that the investigated additives rather did not affect feed taste or, in turn, feed intake [43]. Other reports also confirm that diets supplemented with either phytogenic ingredients or organic acids are well tolerated by the birds and do not compromise performance [44,45,46]. These findings might be considered beneficial since the predictable effect of dietary treatments is the key factor in determining potential dietary intervention for prophylaxis.

## 5. Conclusions

The application of phytogenic products and organic acids to the broilers’ diet had no compromising action on the condition of foot pad. Neither group of additives disturbed the blood indices of metabolism or selected indices for antioxidant status or compromised performance results. Both the investigated additives manifested effects that supported the proliferation, in the caeca, of bacteria that were beneficial to the host, which could contribute to the improvement of the litter quality during the last period of rearing.

## Figures and Tables

**Table 1:** Formulation and nutritional composition of the chickens’ diets.

Item	Starter	Grower I	Grower II	Finisher
Ingredient (%):				
Wheat	35	35.03	36.6	30.12
Maize	24.2	30.00	30.42	38
Soybean meal	0.0	28.6	25	22
Rapeseed meal	3.0	0.0	0.0	2.0
Sunflower oil	1.3	1.9	3.0	3.2
Sunflower meal	0.0	2.0	3.0	3.0
Limestone	1.36	0.91	0.7	0.57
Monocalciumphosphate	0.7	0.38	0.12	0.06
Methionine	0.34	0.28	0.26	0.2
Sulfate of lysine	0.4	0.42	0.42	0.4
NaCl	0.2	0.2	0.2	0.2
Sodium sulfite	0.2	0.2	0.2	0.2
Threonine	0.06	0.08	0.08	0.05
Vitamin−mineral premix ^1^	0.50	0.50	0.50	0.50
Nutritional composition:				
EM * (kcal/kg)	2901.2	3014.5	3108.5	3160.8
Protein (%)	22.36	20.51	19.45	18.43
Fat (%)	3.25	3.94	5.02	5.50

^1^ 1 kg of vitamin−mineral premix contains: Vitamin A, 10,000,000 IU; Vitamin D3, 70,000 IU; HyD, 7500 mcg/kg; Vitamin E, 25,000 mg/kg; Vitamin K3, 600 mg/kg; Thiamine B1, 600 mg/kg; Riboflavin B2, 1600 mg/kg; Pyridoxine B6, 800 mg/kg; Niacin, 10,000 mg/kg; Calcium pantothenate, 3266 mg/kg; Biotin, 40,000 mcg/kg; Choline chloride, 69,565 mg/kg; Zinc, 20,000 mg/kg; Iron, 10,000 mg/kg; Copper, 4000 mg/kg; Iodine, 200 mg/kg; Selenium, 200 mg/kg; Manganese, 20,000 mg/kg. * EM = metabolizable energy.

**Table 2:** Scale of assessment of foot pad dermatitis lesions.

Score	Description
0	No lesions.
1	Superficial lesions, color lesions with diameter not exceeding 0.5 cm.
2	Deep lesions with a scab and ulceration, color lesions with diameter of 0.5 cm or greater.

**Table 3:** Primer sets used for bacteria abundance determination.

Bacteria	Primers	Sequence (5′-3′)	Reference
Total bacteria	Forward	CGTGCCAGCCGCGGTAATACG	Amit-Romach et al. [25]
	Reverse	GGGTTGCGCTCGTTGCGGGAC TTAACCCAACAT	
*Clostridium*	Forward	AAAGGAAGATTAATACCGCATAA	
	Reverse	ATCTTGCGACCGTACTCCCC	Amit-Romach et al. [25]
*Lactobacillus*	Forward	CATCCAGTGCAAACCTAAGAG	
	Reverse	GATCCGCTTGCCTTCGCA	Wang et al. [26]
*Escherichia coli*	Forward	GGGAGTAAAGTTAATACCTTTGCTC	
	Reverse	TTCCCGAAGGCACATTCT	Tsen et al. [27]
*Bifidobacterium*	Forward	CGGGTGCTICCCACTTTCATG	
	Reverse	GATTCTGGCTCAGGATGAACG	Kastner et al. [28]

**Table 4:** Blood biochemical indices in 42-day-old chickens fed experimental diets (*n* = 10).

Indices	Group ^1^	Medium	SE	*p*-Value
ALT (U/L)	C	6.3	0.75	0.101
	A	6.9	0.82	
	P	4.8	0.42	
AST (U/L)	C	422.1	49.3	0.318
	A	508.5	69.79	
	P	399.9	30.8	
Ca (mmol/L)	C	2.51	0.29	0.730
	A	2.68	0.05	
	P	2.53	0.06	
Cl (mmol/L)	C	106.7	2.21	0.179
	A	110.3	0.98	
	P	110.5	1.26	
GGT (U/L)	C	18.30 ^b^	1.25	0.049
	A	22.97 ^a^	1.13	
	P	22.35 ^a^	1.7	
GLU (mmol/L)	C	14.6	0.49	0.377
	A	14.44	0.28	
	P	15.21	0.41	
K (mmol/L)	C	8.02 ^b^	0.96	0.011
	A	5.36 ^a^	0.14	
	P	6.00 ^a^	0.39	
LAC (mmol/L)	C	8.06 ^b^	1.07	0.012
	A	5.24 ^a^	0.51	
	P	5.22 ^a^	0.32	
Mg (mmol/L)	C	0.98	0.19	0.895
	A	0.91	0.02	
	P	0.92	0.04	
Na (mmol/L)	C	148.0	2.34	0.265
	A	149.3	0.96	
	P	152.1	1.66	
P (mmol/L)	C	2.53 ^b^	0.23	0.040
	A	2.13 ^a^	0.04	
	P	2.03 ^a^	0.04	
TP (g/L)	C	30.45	1.44	0.884
	A	30.15	1.16	
	P	29.45	1.72	

^1^ Birds were fed a non-supplemented control diet (C); the control diet supplemented (in addition) with the phytogenic supplement containing hop (A); or the control diet supplemented with organic acids and their salts and prebiotic complex mixed in the proportion of 65/30 *v/v,* respectively (P). ALT, alanine aminotransferase; AST, aspartate aminotransferase; Ca, calcium; Cl, chloride; GGT, gamma-glutamyltransferase; GLU, glucose; K, potassium; LAC, lactate; Mg, magnesium; Na, sodium; P, phosphorus; TP, total protein. a, b: difference between groups is significant at *p* < 0.05.

## Data Availability

All data generated or analyzed during the study are included in this published article. The datasets used and/or analyzed in the current study are available from the corresponding author on reasonable request.
